# Elucidating the effect and mechanism of the brown coal-based amendment on plant availability of zinc, lead and cadmium in a Haplic Luvisols

**DOI:** 10.1007/s11356-021-17424-3

**Published:** 2021-11-22

**Authors:** Alina Maciejewska, Jolanta Kwiatkowska-Malina

**Affiliations:** Department of Spatial Planning and Environmental Sciences, Warsaw University of Technology, Politechniki 1 Sq, 00-661 Warsaw, Poland

**Keywords:** Brown coal-based amendment, Heavy metals, Phacelia, Bio-accumulation index, Haplic Luvisols

## Abstract

Plants are a key link in the trophic chain and therefore may determine the global circulation of pollutants, including heavy metals (HMs). In the context of sustaining soil functions associated with food safety, the bioavailability of HMs should be reduced to a minimum needed for adequate plant nutrition. The objective of the study was to analyse the bioavailability of zinc, lead and cadmium in phacelia *(Phacelia tanacetifolia* Benth*.*) under conditions of varied soil pH and doses of brown coal-based organo-mineral amendment so-called the Rekulter. The experiment was carried out on Haplic Luvisols in field stone pots that sank into the ground, with the following HM content (in mg kg^−1^ of soil): 90.0 (Zn), 60.4 (Pb) and 0.80 (Cd). The Rekulter was applied to the soil in the amounts of 180, 360 and 720 g per pot. The bio-accumulation index (BI) was calculated as a ratio of a HM content in a plant to its total content in a soil sample, and it was used to evaluate bioavailability. The application of the Rekulter reduced the bioavailability of the studied heavy metals: the lowest BI values were found in the case of Pb. The uptake of HMs by phacelia was the smallest for the highest applied Rekulter dose at a soil pH of approximately 6.0. The bioavailability of Zn, Pb and Cd was influenced by soil pH and organic matter content, reducing their mobility and possible environmental risks. The Rekulter reduced HM bioavailability: the lowest bio-accumulation index (BI) values were found in the case of Pb. The application of the Rekulter into soil improved the physical, chemical and biological properties of soil, including the reduction of contaminant bioavailability.

## Introduction

Trace elements, including Zn, Pb and Cd, referred to as heavy metals (HMs) are very important, non-renewable natural resources. The natural content of HMs in soil primarily depends on their abundance in the parent rock, weathering and, soil-forming processes, particle size distribution. Human activities caused the release of excessive amounts of HMs to the environment, to a certain degree causing their incorporation into the food chain. The resulting environmental problem is complex. On the one hand, it involves a considerable loss of ores of which they are components. On the other hand, they occur in increasingly higher concentrations in living organisms and the environment of such organisms (Madejón et al. 2009).

The causes of dispersion and increase in the toxic effect of HMs, on living organisms, are observed over the last 200 years (Wong et al. 2006). Chemical and biochemical transformations, bio-accumulation of HMs and their ability to migrate are of high importance for the state of the environment and human health (Kabata-Pendias and Pendias 2001; Kacprzak and Malina 2005; Zehra et al. 2019; Zhang et al. 2020). HMs have a strong harmful effect on humans, animals and plants (Wu et al. 2017). HMs in soil are highly toxic, persistent and non-biodegradable. They are primarily accumulated in surface horizons of soil, where through plants they are easily supplied to the food chain, and thus determine the global circulation of pollutants, frequently causing different muta- and carcinogenic processes in living organisms (Szewczyk et al. 2007). Plants are a very important link in the trophic chain and therefore may determine the global circulation of pollutants, including HMs. In the context of sustaining soil functions associated with food safety, the bioavailability of HMs should be reduced to a minimum needed for adequate plant nutrition.

In the context of the continuously growing global human population, access to the sufficient amount of safe and nutritious food is of key importance for our life and good state of health (Ikram et al. 2021). Intensive agriculture, industry, urbanisation and developing land transport result in degradation of soils and loss of their basic function, namely biomass production. The rate of anthropogenic transformations of soils rapidly increases with a dynamic population growth and the necessity to meet the growing needs of humanity. This causes a systematic reduction of the surface area of natural soils, an increase in soils polluted with HMs in the global soil resources. Nowadays, the problems that among others Pb contaminates soil have become the subject of current domestic and international attention due to the increasing emission of pollutants caused by industrial production and human activities (Ahmad et al. 2016; Zehra et al. 2019). Therefore, the provision of food safety requires limiting the bioavailability of HMs (Suna et al. 2010).

The transfer of metals from soils to plants depends upon its availability and mobility in soil (Du et al. 2020). Increasing soil sorption capacity that can potentially be achieved by the addition of geosorbents, such as brown coal and brown coal-based amendments to contaminated soil is commonly considered as technically, economically and environmentally effective in situ remediation method to reduce HM bioavailability. Metal cations bind to negatively charged organic compounds, natural and synthetic zeolites (Oste et al. 2002), clay minerals (Usman et al. 2006; Szewczuk-Karpisz et al. 2020) and nanoscale zero-valent iron (Ren et al. 2021). Limiting the availability of HMs for plants and soil biological activity largely depends on soil pH (Hou et al. 2019; Zhong et al. 2021) and soil organic matter (Maciejewska and Kwiatkowska 2000; Skłodowski et al. 2006; Zenga et al. 2011; Ahmad et al. 2016; Kwiatkowska-Malina and Maciejewska 2013; Wang et al. 2020). The bioavailability of Cd increased at pH > 5.5, for Zn, Ni and Cu at pH 5.0 and at pH < 4.5 in the case of Pb (Blake and Goulding 2002). At acidic pH, H^+^ ions compete at metal binding sites which cause an increase in free metal ions that become available to plants (Sandrin & Hoffman 2007). Zn, Cd, Cr and Pb are absorbed readily from the soil at acidic pH (Blaylock & Huang 2000). A soil with relatively low organic matter content has a greater risk of metal contamination. Soil organic matter influences cation exchange capacity (CEC), buffering capacity and retention capacity of metals in soil by forming stable metal chelates. Therefore, metals in mineral soils are more mobile and bioavailable compared to organic soils (Balasoiu et al. 2001).

A potential source of organic matter in soils can be unconventional soil amendments (Maciejewska and Kwiatkowska 2000; Kwiatkowska et al. 2006; Liu et al. 2018a, b; Amoah-Antwi et al. 2020). Generally, amendments with processed and stable organic materials can transform metals in contaminated soil from their exchangeable forms to more stable organic phases (Smith 2009; Guo et al. 2018). Negatively charged functional groups, which are integral components of organo-metallic complexation, progressively increase with humification and thus are abundant in brown coal-derived humic substances in soil (Turgay et al. 2011). These properties, together with the highly porous structure of brown coal, can increase soil pH, CEC and specific surface area (SSA), hence improving the metal sorption capacity of the soil (Kwiatkowska et al. 2006).

Excess uptake of trace elements from polluted soils by plants is phytotoxic (Malina 2004; Zhou et al. 2016). Reducing the HM solubility and bioavailability without removing them from contaminated soils is one measure that could weaken detrimental HMs impacts on the environment (Richards et al. 2000). The negative effect of HMs can be mitigated through a change of soil conditions, often involving immobilisation of HMs which does not lead to a decrease in their total content in the soil, but efficiently reduce the bioavailability and thus ecological risk (Basta et al. 2005). Factors facilitating bioavailability of HMs primarily include very high concentration of a given metal in soil and acidic soil pH (Blake and Goulding).

Due to the still high level of mobilisation of HMs in the environment; the dynamics of transformations of urban, industrial and agricultural areas and the undoubtedly negative effect of the pollutants on human health, complex research works concerning the issue are fully justified. This paper considers the use of brown coal-based organo-mineral amendment so-called the Rekulter as a potential, inexpensive and natural tool in mitigating/remediating HMs contaminated soil. More specifically, the objective of the study was to analyse the content of zinc, lead and cadmium in phacelia (*Phacelia tanacetifolia* Benth.) under conditions of varied soil pH and doses of the Rekulter.

## Materials and methods

### Experimental design and organo-mineral amendment

The experiment was carried out in Skierniewice (Poland) (20°34′E, 51°58′N) in field stone pots (sunk in the ground) with a diameter of 40 cm and height of 120 cm, filled with soil with different pH (around 4.0, 5.0 and 6.0), collected with preservation of natural genetic levels in the profile, Haplic Luvisols, according to the World Reference Base for Soil Resources (IUSS Working Group WRB 2015) developed from heavy loamy sand on light loam containing in Ap horizon (layer of 0–25 cm) the following fractions: sand (> 0.05 mm), 87%; silt (0.02–0.05 mm), 6% and clay 7% (< 0.02 mm), to the amount of 56 kg per pot. The abovementioned soil initially was characterised as follows: C_total_ organic carbon (C_tot_), 7.62 g kg^−1^; N_total_ (N_t_), 0.6 g kg^−1^; C_tot_:N_t_ ratio was 12.3; P available, 73.4 mg kg^−1^; K available, 91.1 mg kg^−1^.

The Rekulter is an organo-mineral amendment obtained from brown coal contained: 85% brown coal, 10% lowmoor peat, 4% brown coal ash and 1% mineral fertilisers (potassium salt (50% K_2_O), 0.4%; urea (46% N), 0.4%; simple superphosphate (19% P_2_O_5_), 0.2%). As the basic component of the Rekulter, brown coal is used in the form of the so-called sand-gravel aggregate of a particle size less than 2 mm, which is a side product in coal mining. The brown coal was not contaminated with heavy metals. The particular components (mentioned above) were mixed and pulped. Selected chemical composition (in % dry matter) of the Rekulter: C 62, Ca 8, N 0.68, P 0.50, K 0.25, Mg 0.50. The Rekulter was introduced to the soil once and mixed with the entire soil mass in the pots, at doses of 180 (dose 1), 360 (dose 2) and 720 (dose 3) g per pot, respectively 14, 28 and 56 t per ha, corresponding to 5 t, 10 t and 20 t C_org_ per ha. The application doses of the Rekulter were based on equal amounts of organic carbon content introduced into the soil with farmyard manure. Soil without amendment was the control treatment. The soil was also supplied with heavy metals (HMs) in the following quantities in mg kg^−1^: Zn, 90 in the form of ZnSO_4_ 7 H_2_O; Pb, 60 in the form of Pb(NO_3_)_2_ and Cd, 0.8 in the form of Cd(NO_3_) 4 H_2_O. The soil was characterised by higher than average content of heavy metals (I degree, evaluated concentration in mg kg^−1^ of dry soil mass: Zn, 50–100; Pb, 30–70; Cd, 0.3–1.0 (Kabata-Pendias et al. 1993). In the experiment, due to the short growing season at different times in order to bloom at the desired time phacelia (*Phacelia tanacetifolia* Benth.), a honey plant was cultivated. Phacelia was sown in July and harvested in September during the blooming phase. The following average doses of nutrients were applied: N, 190; P, 46; K, 175 kg ha^−1^. The average annual temperature and precipitation are 8 °C and 520 mm, respectively.

### Soil analysis

Before the experiment was set up, soil samples were assayed for pH in 1 M KCl (1:2.5 w/v) by the potentiometric method. Soil samples for analysis were taken from randomly chosen locations in the stone pot from the 0–20-cm horizon using a soil sampling rod, after plant harvest, air-dried, mixed, sieved (ø 2 mm) and analysed. Soil samples were mineralised in a mixture of concentrated acids HCl + HNO_3_ (3:1 + 30% H_2_O_2_) were subject to the determination of cadmium, lead and zinc. The determination of bioavailable forms of zinc, lead and cadmium in soil involved the application of selective extraction by means of 0.05 M solution of EDTA (0.017 M EDTAH_4_ + 0.01135 M Ca(CH_3_COO)_2_ · 2 H_2_O + 0.019 M C_3_H_4_(OH)(COOH)_3_ H_2_O + NH_3_, pH 7.3) (Ure 1996).

### Plant analysis

Plants were collected and washed, weighted, cut into pieces and dried at 60 °C to constant weight. Then, 1.0- to 2.0-g subsamples were ground in a stainless steel mill and mineralised in a liquid mixture of concentrated acids (HNO_3_ to HClO_4_, volumetric ratio of 4:1). Content of lead, zinc and cadmium in plant samples were determined with ICP-AES.

The bio-accumulation index (BI) was calculated as a ratio of a heavy metal content in a plant to its total content in a soil sample, and it was used to evaluate the mobility of lead, zinc and cadmium in the soil, as well as their availability to plants. Mathematically, BI is expressed as

$$\mathrm{BI}={\mathrm{C}}_{\mathrm{plant}}\mathrm{ tissue}/{\mathrm{C}}_{\mathrm{soil}}$$

where C_plant_ tissue is the content of metal in plant tissue in milligram per kilogram dry mass of plants. C_soil_ is the content of the metal in the soil in milligram per kilogram dry mass of soil.

### Statistical analysis

The results of the experiment were carried out in triplicate. For all data processing, a standard statistical software package Statgraphics 4.1 was applied. One-way ANOVA analyses were undertaken to establish significant differences (*p* < 0.05) between the mean values of different treatments.

## Results and discussion

### Plant yields

The mean biomass (fresh and dry mass) of phacelia *(Phacelia tanacetifolia* Benth.) is presented in Table 1. The Rekulter positively affected the growth of phacelia biomass. The highest (533 g) fresh mass yield of phacelia was obtained in the variant with the highest Rekulter dose on slightly acidic soil. The lowest fresh mass yield (240 g) of phacelia was obtained in the control on strongly acidic soil. The same dependences occurred for the dry mass yield of phacelia: 132 g in the variant with the highest dose of the Rekulter on slightly acidic soil and 52 g in the control variant on strongly acidic soil (pH KCl = 4.0), respectively.

**Table 1:** Biomass of phacelia (*Phacelia tanacetifolia* Benth) (fresh and dry mass)

Rekulter dose (g pot^−1^)	pH	Fresh mass (g pot^−1^)	Dry mass (g pot^−1^)
Control ‘0’	6.0	461	102
	5.0	323	66
	4.0	240	52
180	6.0	487	115
	5.0	451	100
	4.0	333	68
360	6.0	481	111
	5.0	382	94
	4.0	367	68
720	6.0	533	132
	5.0	470	108
	4.0	422	87
LSDα = 0.05	-	10.9	7.3

*LSD*, least significant difference

An increase in the biomass of phacelia with the dose of the Rekulter introduced to the soil resulted from the fact that it was a source of macro- and microelements for the cultivated plants. It is in accordance with the study results obtained earlier by the authors (Kwiatkowska-Malina and Maciejewska 2013; Maciejewska and Kwiatkowska-Malina 2019). Coal is an extremely heterogeneous porous material containing both organic macerals and inorganic mineral matter. The macromolecule structure of coal macerals offers plenty of adsorption sites for gaseous and liquid adsorbates (Liu et al. 2018a, b). Due to properties of brown coal such as porosity and specific surface area, the Rekulter shows high CEC not only towards the water, but also towards macro- and microelements (Kwiatkowska et al. 2006). Therefore, it has a buffer effect on soil pH and nutrient concentration in the soil solution, providing better conditions for plants growth. Organic and mineral fertilisation has a determining role in shaping yields and chemical composition of plants (Nardi et al. 2004), as also confirmed by the obtained study results.

### Heavy metals accumulation in phacelia

The introduction of the Rekulter to the soil contributed to a decrease in the content of Zn, Pb and Cd in the dry mass of phacelia. Mean contents of the analysed heavy metals (HMs) are presented in Table 2. The content of Zn in phacelia was the highest (125 mg kg^−1^ d.m.) in the control variant on strongly acidic soil (pH KCl = 4.0) and the lowest (72 mg kg^−1^ d.m.) in the variant with the highest Rekulter dose (720 g) on slightly acidic soil (pH KCl = 6.0). Pb content in phacelia was the highest (17.1 mg kg^−1^ d.m.) in the control variant on strongly acidic soil. In the variant with the highest Rekulter dose on slightly acidic soil, Pb content in phacelia decreased by approximately 50% in comparison to plants from the control variant. Considering critical values for Pb adopted at a level below 1.0 mg kg^−1^ d.m. of consumption plants (Kabata-Pendias et al. 1993), it was determined that phacelia neither the control variant nor variants with an addition of the Rekulter can be used by bees due to the occurrence of the dependency of Pb content in honey on its content in honey plants. Also, in the case of Cd in phacelia, the content of it decreased with the addition of the Rekulter to the soil. Cd content in phacelia was the lowest (0.45 mg kg^−1^ d.m.) in the variant with the highest Rekulter dose on slightly acidic soil. In the variant with the highest Rekulter dose on slightly acidic soil, Cd content in phacelia decreased by approximately 60% in comparison to the control variant. Phacelia had no consumption value in terms of content (above 0.1 mg kg^−1^ d.m. of plants) of Cd from any variant irrespective of soil pH or the Rekulter dose.

**Table 2:** Contents of Zn, Pb and Cd in phacelia *(Phacelia tanacetifolia Benth)* (mg kg^−1^ d.m.)

Rekulter dose (g pot^−1^)	pH	Zinc (Zn)	Lead (Pb)	Cadmium (Cd)
Control ‘0’	6.0	118	14.3	1.27
	5.0	120	15.7	1.29
	4.0	125	17.1	1.31
180	6.0	95	9.9	0.48
	5.0	102	9.2	0.51
	4.0	105	10.9	0.60
360	6.0	87	94	0.46
	5.0	91	11.2	0.50
	4.0	97	11.7	0.57
720	6.0	72	9.1	0.45
	5.0	80	9.7	0.47
	4.0	94	10.5	0.55
LSDα = 0.05	-	7.1	3.14	0.039

*d.m.*, dry mass; *LSD*, least significant difference

The amount of metals subject to uptake by plants depends on the type of metals, their content in the soil, forms of their occurrence and crop species (Brunetti et al. 2009). The main soil factors having an impact on the mobility of HMs and the total content in the soil are the CEC, soil pH, mechanical composition of the soil, humus content and the interaction among them (Angelova et al. 2009). An increase in the content of organic matter in mineral soils, with very low content of humic substances is of high importance for limiting uptake of HMs by plants (Maciejewska and Kwiatkowska 2000). Organic matter, binding HMs into compounds insoluble or hardly soluble in water, limits their desorption to the soil solution and therefore their availability for plants (Kabata-Pendias and Pendias 2001). The durability of complex ‘organic matter-metal’ bonds increased with an increase in the degree of its humification and a decrease in soil acidity (Senesi 1992). It is related to the immobilisation of HMs by micromolecular organic mineral colloids and to the general improvement of physicochemical properties of soils, as also reflected in an increase in phacelia yield. Brown coal, constituting the primary component (85%) of the Rekulter, as well as products of its humification in soil, can develop complex compounds with HMs with varying durability. The highest durability usually concerns complexes with Cu and Pb, followed by Ni, Cd and Zn. The obtained results are confirmed by studies by other authors (Gode and Pehlivan 2005), where the introduction of brown coal to the soil caused a decrease in the content of HMs in plants. In general, the HM contents in phacelia have been decreased, so the ecological risk of entering the food chain was reduced.

The Rekulter contributed to a decrease in the phytoavailability of HMs and their uptake by phacelia. The concentration of HMs in plants largely depends on the species or even cultivar of plants (Zhou et al. 2016). Varied sensitivity of plants to the availability of Cd and Zn in the soil permits selection of plants able to grow in concentrations toxic for other plants (Richards et al. 2000; Brunetti et al. 2009). According to Sauerbeck (1991), dicotyledonous plants absorb metals considerably easier than monocotyledonous plants. Zn is usually absorbed by plants proportionately to its content in the soil, although both soil properties and selection of species considerably affect its accumulation in plants. Pb uptake by plants depends on soil properties, characteristic features of the species and physiological state of the plant. Considering the critical values for Pb adopted at a level of less than 1.0 mg kg^−1^ d.m. of plants (Kabata-Pendias et al. 1993), it was determined that phacelia from any variant can be used by bees due to the occurrence of the dependency of Pb content in honey and its content in the entomophilic plant (Rowarth 1990). Heavy metals are the most toxic for plants and carcinogenic for people include Cd (Zhou et al. 2016). Also, in the case of Cd, phacelia from any variant, irrespective of soil pH and the Rekulter dose, had any consumption value in terms of Cd content (below 0.1 mg kg^−1^ d.m. of plants). Evangelou et al. (2007) reported that lacy phacelia is possible to be suitable for phytoextraction for Pb and Cd at low and moderately contaminated sites. Higher than normal content of Cd in honey plants can also result in the exceedance of acceptable values of Cd content in bee products (Cesco et al. 1994). High quality of bee products and health of their consumers, but also continuously decreasing bee population, and consequently decreasing biodiversity depends on among others the content of HMs in honey plants such as phacelia.

### Residual heavy metals in soil

The introduction of the Rekulter to the soil had no considerable effect on the content of ‘total’ forms of Zn, Pb and Cd (HCl + HNO_3_ (3:1 + 30% H_2_O_2_)) in the soil, but the content of the bioavailable form (0.05 M EDTA) of the analysed HMs significantly decreased (Table 3).

**Table 3:** ‘Total’ (HCl + HNO_3_ (3:1 + 30% H_2_O_2_)) and bioavailable (0.05 M EDTA) contents of Zn, Pb and Cd in soil (mg kg^−1^ d.m.)

Rekulter dose (g pot^−1^)		‘Total’			Bioavailable		
	pH	Zn	Pb	Cd	Zn	Pb	Cd
Control ‘0’	6.0	89.9	50.9	1.09	71.7	37.8	0.53
	5.0	89.9	50.9	1.09	71.9	37.8	0.53
	4.0	79.5	50.3	1.09	71.5	41.8	0.51
180	6.0	80.5	37.6	0.79	55.5	24.2	0.45
	5.0	81.7	34.0	0.78	52.9	21.3	0.40
	4.0	77.2	39.0	0.82	57.2	25.2	0.41
360	6.0	80.3	35.3	0.79	52.8	27.5	0.45
	5.0	83.8	40.2	0.76	56.2	26.3	0.41
	4.0	79.3	35.3	0.78	57.3	26.2	0.41
720	6.0	80.0	37.2	0.80	47.1	24.6	0.38
	5.0	77.5	36.6	0.78	49.2	24.1	0.34
	4.0	78.2	38.3	0.78	43.2	23.1	0.31
LSDα = 0.05	-	7.23	3.43	0.121	3.33	2.81	0.092

*d.m.*, dry mass; *LSD*, least significant difference

Contents of bioavailable forms of Zn, Pb and Cd in the soil from the control variant were from 71.5 to 71.9; from 37.8 to 41.8 and from 0.51 to 0.53 mg kg^−1^ d.m. of soil, respectively, in reference to ‘total’ forms constituting: from 79 to 89%; from 74 to 84% and from 46 to 49%. In the variant with the highest Rekulter dose, contents of a bioavailable form of Zn, Pb and Cd in the soil were from 43.2 to 47.1; from 23.1 to 24.6 and from 0.31 to 0.38 mg kg^−1^ d.m. of soil, respectively, in reference to the content of ‘total’ forms constituting from 55 to 63%; from 60 to 66% and from 40 to 47%, respectively. The effect of HMs to a low degree depends on the content of their ‘total’ forms in the soil. It is primarily determined by their bioavailability, including factors such as physicochemical soil properties and the content of organic matter. Treatments aimed at immobilisation of HMs in the soil, including the application of organic mineral amendments with high SSA and CEC do not lead to a decrease in the content of their ‘total’ forms, but limit their bioavailability, considerably limiting the ecological risk (Basta et al., 2005; Wang et al. 2020). The addition of the Rekulter contributes to the adsorption of HMs by soil humus components, inhibiting the migration of HMs in soil-phacelia systems to reduce the HM accumulation in phacelia. The Rekulter contributed to a decrease in the content of the bioavailable form of the HMs. A decrease in the content of bioavailable forms of HMs in the soil was possible due to the occurrence in brown coal of functional groups such as -NH, -OH, -CH and -COO that can develop complexes with HMs (Liu et al. 2018a, b). It is in accordance with earlier study results by the authors (Maciejewska and Kwiatkowska 2000; Kwiatkowska-Malina and Maciejewska 2013). Also, Zenga et al. (2011) point out that the phytoavailability of HMs depends among others on soil properties such as soil pH and content of organic matter.

### Bio-accumulation indices

**Fig. 1:**
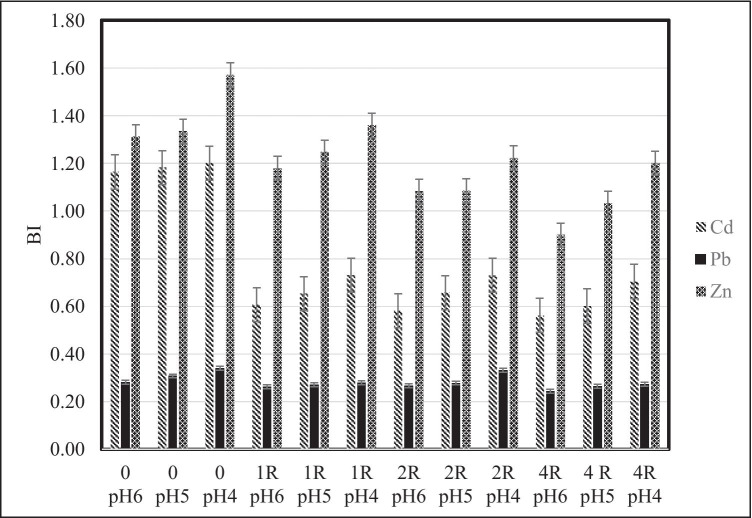
Bio-accumulation indices (BI) of Zn, Pb and Cd

Values of the bio-accumulation index (BI) for Zn, Pb and Cd were the highest for phacelia cultivated on very acidic soil, both in the control variant, and on that with an addition of the Rekulter, irrespective of the dose (Fig. 1).

BI was calculated as a ratio of a heavy metal content in the plant to its ‘total’ content in soil (least significant difference (LSD)).

BI values for Zn after the application of the highest dose of the Rekulter were the lowest and varied from 0.90 to 1.20. In the case of Pb, BI values were approximate on all variants irrespective of soil pH and the Rekulter dose. The highest BI value for Pb, i.e. 0.24 occurred after the application of the highest dose of the Rekulter on slightly acidic soil. The BI value for cadmium after the application of the highest Rekulter dose varied from 0.56 (slightly acidic soil) to 0.71 (very acidic soil). The BI determines the plant’s potential for accumulation of elements with consideration of their initial content in the soil (Skłodowski et al. 2006; Zhou et al. 2016). The BI values differed significantly for metals. Butt et al. (2018) reported that they recorded the highest average bio-accumulation factor (BAF) for Zn (3.35) and the least for Cd (1.22) with intermediate values for Pb (2.94). The higher values are adopted by BI, the higher concentration of the element is determined in the plant biomass in reference to the initial values in soil. BI values for Zn, Pb and Cd were the highest for phacelia cultivated on strongly acidic soil both on the control and with the addition of the Rekulter, irrespective of the dose.

## Conclusions

In this work, a novel brown coal waste-based organo-mineral amendment so-called the Rekulter was used to reduce the bioavailability and bio-accumulation of Zn, Pb and Cd for phacelia (*Phacelia tanacetifolia* Benth.) under varied soil pH and doses, and it was found that the Rekulter has high fertilisation value expressed in phacelia biomass. The bioavailability of Zn, Pb and Cd was influenced by soil pH and organic matter content, reducing their mobility and possible environmental risks. The Rekulter contributed to a decrease in the bioavailability of Zn, Pb and Cd, and therefore, their uptake by phacelia was considerably lower in comparison to plants from the control variant. The determined content of Cd, Pb and Zn in phacelia biomass was higher than threshold values for acceptable contents of heavy metals (HMs) in plants for consumption. The Rekulter reduced HMs bioavailability: the lowest bio-accumulation index (BI) values were found in the case of Pb. The uptake of Zn, Pb and Cd by phacelia was the lowest for the highest applied Rekulter dose at a soil pH of approximately 6.0. The application of the Rekulter into the soil to improve the physical, chemical and biological properties of soil, including the reduction of contaminant bioavailability is very important area of research.

## Data Availability

The datasets used and analysed during the current study are available from the corresponding author on reasonable request.
